# Piperlongumine Attenuates High Calcium/Phosphate-Induced Arterial Calcification by Preserving P53/PTEN Signaling

**DOI:** 10.3389/fcvm.2020.625215

**Published:** 2021-02-10

**Authors:** Wenxiang Shi, Jieyu Lu, Junhan Li, Ming Qiu, Yan Lu, Jia Gu, Xiangqing Kong, Wei Sun

**Affiliations:** ^1^Department of Cardiology, The First Affiliated Hospital of Nanjing Medical University, Nanjing, China; ^2^Department of Translational Medicine, Collaborative Innovation Center for Cardiovascular Disease Translational Medicine, Nanjing Medical University, Nanjing, China

**Keywords:** piperlongumine, P53, PTEN, vascular calcification, VSMCs, STAT3

## Abstract

Vascular calcification frequently occurs in the process of chronic kidney disease, atherosclerosis and aging, resulting in an increased prevalence of cardiovascular events. Piperlongumine (PLG) is a natural product isolated from *Piper longum* L. Here, we aimed to explore the effect of PLG in high calcium- and phosphate-induced vascular calcification and the associated mechanism. Flow cytometry assays showed that PLG at concentrations <10 μM did not promote vascular smooth muscle cells (VSMCs) apoptosis, and PLG at concentrations >2.5 μM inhibited VSMCs proliferation. Thus, 2.5 μM PLG was selected for subsequent experiments. Alizarin red staining and ALP activity assays showed that PLG inhibited calcium deposition of VSMCs treated with high calcium/phosphate medium. PLG also decreased the expression of osteogenic genes and proteins, including Runx2, Bmp2, and OPN, as determined by qRT-PCR and western blotting. In a vitamin D-induced aortic calcification mouse model, a 5 mg/kg dose of PLG decreased calcium deposition in the aortic wall as well as Runx2 expression. With regard to the mechanism, we found that the levels of P53 mRNA and protein in both VSMCs and mouse aortic tissues were decreased in the calcification models, and we observed that PLG preserved the levels of P53 and its downstream gene PTEN. Concurrent treatment of VSMCs with P53 ShRNA and PLG blunted the anti-calcific effect of PLG. In conclusion, PLG attenuates high calcium/phosphate-induced vascular calcification by upregulating P53/PTEN signaling in VSMCs. PLG may act as a promising herbal extract for the clinical management of vascular calcification.

## Introduction

Vascular calcification is a complex disease that can occur in large and small blood vessels throughout the body. The main feature of vascular calcification is the deposition of calcium-containing complexes along the blood vessel wall. These deposits are mainly composed of calcium and phosphate minerals in the form of hydroxyapatite crystals, which are similar to those in bone tissue (1). The appearance of ectopic hydroxyapatite in blood vessels indicates that vascular calcification is forming. Vascular calcification has been previously considered to be a passive and degenerative process due to the imbalance of calcium and phosphorus metabolism. Vascular calcification is now recognized as an active biological process that shares many features with physiological bone formation (2). There are many causes of vascular calcification, including diabetic angiopathy, chronic kidney disease, lipid metabolism disorders, and genetic factors (3). Currently, no theory completely explains the pathogenesis of vascular calcification, and no specific treatment methods for vascular calcification are preferred. Therefore, the search for effective treatment methods for vascular calcification is of great significance for the future protection of human cardiovascular health. Vascular smooth muscle cells (VSMCs) are thought to constitute the main cell type in vascular calcification, and they play important roles in vascular calcification. VSMCs exhibit a contraction phenotype in normal adult blood vessels. However, the phenotype of vascular smooth muscle can be transformed according to changes in the surrounding environment. Previous studies have shown that VSMCs can transform and exhibit a series of cell phenotypic characteristics, including those of osteoblasts, chondrocytes, adipocytes, and macrophage foam cells. In calcified blood vessels, VSMCs show osteogenic differentiation, that is, transformation from a contractile phenotype to a bone/cartilage mineralized phenotype, which is characterized by the development of calcified vesicles, downregulation of mineralization-inhibiting molecules, and increased calcified matrix (elaboration of a calcification-prone matrix) (4). This transformation is accompanied by loss of the smooth muscle cell marker smooth muscle 22 alpha (SM22α) and increase in osteochondrocyte markers, including runt-related transcription factor 2 (Runx2), bone morphogenetic protein 2 (Bmp2), osteopontin, osteocalcin, and alkaline phosphatase (ALP). Overexpression of Bmp2 in vascular smooth muscle cells increases the level of calcification, and Bmp2 expression is increased in the calcified atherosclerotic plaques of blood vessels (5). These findings suggest that Bmp2 is involved in the occurrence and development of vascular calcification. Osteogenic cells lacking Runx2 cause osteogenic dysfunction. Mice with a homozygous mutation of Runx2 die after birth because of abnormal tracheal cartilage (6). VSMC-specific suppression of Runx2 expression inhibits vascular calcification (7). Therefore, Runx2 can be used as an early marker of smooth muscle cell osteoblast differentiation and vascular calcification. ALP is an early indicator of extracellular matrix deposition and plays a key role in promoting bone mineralization. An increase in ALP activity often indicates the occurrence and development of vascular calcification (8, 9).

Piperlongumine (Piplartine,(E)-1-(3-(3,4,5-Trimethoxyphenyl)acryloyl)-5,6-dihydropyridin-2(1H)-one) is a cell-permeable, orally bioavailable natural product isolated from the *Piper longum* L. plant species. The reported pharmacological activities of PLG include anti-inflammatory, antibacterial, anti-atherosclerotic, antioxidant, antitumour, antiangiogenic and anti-diabetic activities. In various types of cancer cells, PLG significantly enhances the expression of wild-type P53 and PUMA, and it inhibits the expression of many pro-survival proteins, such as BCL2, survivin and XIAP (10). With regard to the cardiovascular aspect, studies have shown that PLG treatment reduces the formation of atherosclerotic plaques in mice and inhibits PDGF-BB-induced proliferation of VSMCs *in vitro* (11). PLG can also inhibit the proliferation, migration and ECM expression of smooth muscle upon inactivation of the ERK1/2 signaling pathway, thereby improving cardiac fibrosis (12). However, the functional role of PLG in vascular calcification and relevant osteogenic differentiation has not yet been clarified. In this study, we determined the effect of PLG on high calcium- and phosphate-induced vascular calcification, and we further explored its potential molecular mechanisms.

## Materials and Methods

### Cell Culture

Rat vascular smooth muscle cells (RVSMCs) were isolated by collagenase digestion and cultured in Dulbecco's modified Eagle's medium (Gibco; Thermo Fisher Scientific, Inc., Waltham, MA, USA) supplemented with 15% fetal bovine serum (ScienCell Research Laboratories, Inc., San Diego, CA, USA), 100 U/ml penicillin, and 100 μg/ml streptomycin. Normal RVSMCs from passages three to five were used for subsequent experiments. RVSMCs were cultured in calcification medium containing 1.5 mM calcium and 2 mM phosphate for 3 days to induce calcification as described previously (13, 14). For PLG treated groups, 2.5 μM PLG was added into medium or calcification medium and cells were cultured for 3 days.

### Antibodies and Reagents

Antibodies targeting alpha-smooth muscle actin (α-SMA), smooth muscle 22 alpha (SM22α), and bone morphogenetic protein 2 (Bmp2) were purchased from Abcam (Cambridge, USA). The antibodies targeting runt-related transcription factor 2 (Runx2), phospho-Smad1/Smad5 (p-Smad1/5), phosphatase and tensin homolog (PTEN), and GAPDH were purchased from Cell Signaling Technology, Inc. (Danvers, USA). The antibody targeting osteopontin (OPN) was purchased from Proteintech Group, Inc. (Rosemont, USA). The antibody targeting P53 was purchased from Proteintech Group, Inc. (Minneapolis, USA). Piperlongumine (PLG, SML0221) and vitamin D were obtained from Sigma-Aldrich (Merck KGaA, Germany). The purity of PLG was ≥ 97% (HPLC). The empirical formula (Hill notation) of PLG is C_17_H_19_NO_5_, and the chemical structure of PLG is shown as follows:


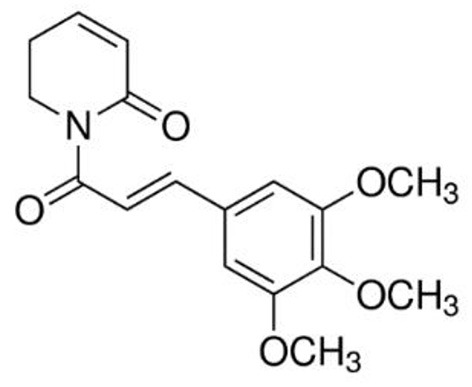


### Animal Experiments

All procedures of this experiment were performed in accordance with animal protection guidelines and were approved by the Laboratory Animal Care and Use Institutional Committee. In total, 35 specific pathogen-free (SPF) male C57BL/6 mice (8 weeks old and 23–26 g) were provided by Vital River Laboratories. To induce vascular calcification, the animals were intraperitoneally injected with vitamin D (Vit D; 8.75 mg/kg/day) or olive oil for 14 days. In addition, mice were subcutaneously injected with PLG (5 mg/kg every 2 days) or vehicle for 28 days starting at the time of the Vit D injection. The mice were housed in plastic cages with a light/dark cycle of 12/12 h and had free access to water and a basal diet. The mice were anesthetized with 2% isoflurane to collect blood for serum calcium assays. Following phosphate-buffered saline (PBS) perfusion, tissues were collected and stored under specific conditions.

### Flow Cytometry

RVSMCs were seeded in 6-well culture plates at a density of 20,000 cells/well. Two days after being seeded, the cells were treated with PLG (1, 2.5, 5, 10, 15, or 20 μM) for 24 h. The cells were then centrifuged at 450 × g for 5 min at 4°C. The subsequent procedures were performed as previously described (1). The results are presented as the percentage of positively stained cells/total cells.

### Real-Time Cell Analysis (RTCA)

RVSMCs proliferation was measured in real-time using an xCELLigence system (Roche Applied Science, Penzberg, Germany). Cells were seeded at a density of 2,000 cells per well and allowed to attach for 12 h. After 24 h, the cells were incubated with piperlongumine (1, 2.5, 5, 10, 15, or 20 μM), or DMSO solvent (2 μl/ml) at 37°C in 5% CO_2_. The cell index at each time point (0, 1, 6, 12, 18, 24, 30, 36, 42, 48, 54, 60, 66, 72, 78, 84, 90, and 96 h) was normalized to the value recorded at time point 0 (baseline).

### Real-Time Polymerase Chain Reaction

Total RNA of RVSMCs and mouse aortas was extracted using TRIzol reagent (Invitrogen, Thermo Fisher Scientific, Inc.), and a PrimeScriptTM RT reagent kit (TaKaRa) was used to perform the reverse transcriptase reaction. Real-time PCR was performed on an ABI Prism 7,900 system. The mRNA expression of the Runx2, OPN, Bmp2, and P53 target genes was normalized to the endogenous expression of GAPDH; the results are presented as fold-change relative to the control. The primers used for real-time PCR to amplify the target genes were as follows: rat Runx2 forward, 5′ — TCTCAGATCGTTGAACCTTGCTA-3′ and reverse, 5′ — TGGTTACTGTCATGGCGGGTA-3′; rat Bmp2 forward, 5′ — ATTAGCAGGTCTTTGCACCAAGAT-3′ and reverse, 5′ — CCCTCCACAACCATGTCCTGA-3′; rat OPN forward, 5′ — GACACGAAGGTAAAGGTGAC-3′ and reverse, 5′ — CTGGTGCTCGTCCTCTACTAC-3′; rat P53 forward, 5′ — ATCTGGACGACAGGCAGACT-3′ and reverse, 5′ — TCCAGCGTGATGATGGTAAG-3′; rat GAPDH forward, 5′ — GGCACAGTCAAGGCTGAGAATG-3′ and reverse, 5′ — ATGGTGGTGAAGACGCCAGTA-3′; mouse P53 forward, 5′ — CTCTCCCCCGCAAAAGAAAAA-3′ and reverse, 5′ — CGGAACATCTCGAAGCGTTTA-3′. TaqMan primers and probes were used for real-time PCR detection of Runx2 (sense primer, 5′-CTTTTGGGATCCGAGCAC-3′; antisense primer, 5′-GGCTCACGTCGCTCATCT-3′; probe, Roche UPL #66) and OPN (sense primer, 5′-AATCTAAGAAGTTCCGCAGATCC-3′; antisense primer, 5′ — CCACATGTGACGTGAGGTCT-3′; probe, Roche UPL #3). Fold differences were calculated for each group using normalized CT values.

### Alizarin Red Staining

Cells were washed three times with Ca^2+^-free PBS, fixed with 4% paraformaldehyde for 10 min, and then dehydrated with 95% ethanol for 20 min. The cells were then stained in 1% Alizarin red solution (pH 4.2; cat. no. BM1853; Hefei Bomei Biotechnology; Hefei, China) for 2 min to visualize the matrix calcium deposition. The remaining dye was washed out with distilled water, and the samples were photographed.

### Calcium Quantitation

Calcium deposition was measured by the o-cresolphthalein complexone method using a QuantiChrom^TM^ calcium assay kit (cat. no. DICA-500; BioAssay Systems). Matrix calcium deposition was fixed with 0.6 M HCl overnight at 4°C and extracted from cells. The following procedures were performed as previously described (1).

### Alkaline Phosphatase Activity Assay

ALP activity was analyzed using a LabAssayTM ALP kit (cat. no. 291–58601; Wako Chemicals Gmbh) in accordance with the manufacturer's protocol. Cells were lysed with 250 μl of 0.05% Triton X-100 in PBS at 4°C. The cell lysates were collected after being frozen and thawed three times. Supernatants (20 μl) were mixed with ALP reagent (100 μl), and the ALP activity was estimated after incubation at 37°C for 15 min. The absorbance was measured at 405 nm and compared to a standard curve to calculate the ALP activity (U, μmol p-nitrophenyl phosphate released per min). The ALP activity was normalized to total protein content.

### Western Blot Analysis

Target proteins extracted from cultured cells and mouse tissues were evaluated by western blot analysis as previously mentioned. Protein concentration was measured using a BCA protein assay kit (Thermo Fisher Scientific; No. 23225) according to the manufacturer's instructions. Protein samples (30 μg) were dissociated by 10~15% SDS–polyacrylamide gels and transferred onto polyvinylidene difluoride membranes (Millipore). After blocking with Tris-buffered saline containing 0.1% Tween 20 and 5% bovine serum albumin for 2 h at room temperature, the membranes were incubated with primary antibodies overnight at 4°C. Membranes were then washed three times with TBST for 10 min and incubated with horseradish peroxidase-labeled secondary antibodies (1:5,000 dilution) for 2 h at room temperature.

### Immunohistochemistry and Immunofluorescence

After being isolated from mice, aortic tissues were embedded in a paraffin block, and paraffin sections were prepared. Paraffin sections were deparaffinized and then subjected to antigen retrieval. Sections were incubated with a blocking solution (10% horse serum, 0.05% Triton X-100, and 5% bovine serum albumin) for 1 h to reduce non-specific background staining. Nuclei were detected by DAPI staining for 20 min. Tissue sections were overlaid with cover slips and observed by laser scanning microscopy (Zeiss).

### Statistical Analysis

All experiments were performed at least three times. All experiment data are expressed as the means ± standard error. Treatment group values were compared with corresponding control values using GraphPad Prism 6.0 (GraphPad software). Statistical significance was determined by one-way analysis of variance (ANOVA) followed by Bonferroni's multiple comparison test. A *P* < 0.05 indicated a statistically significant difference.

## Results

### Piperlongumine at Low Concentration Does Not Affect the Proliferation and Apoptosis of VSMCs

**Figure 1:**
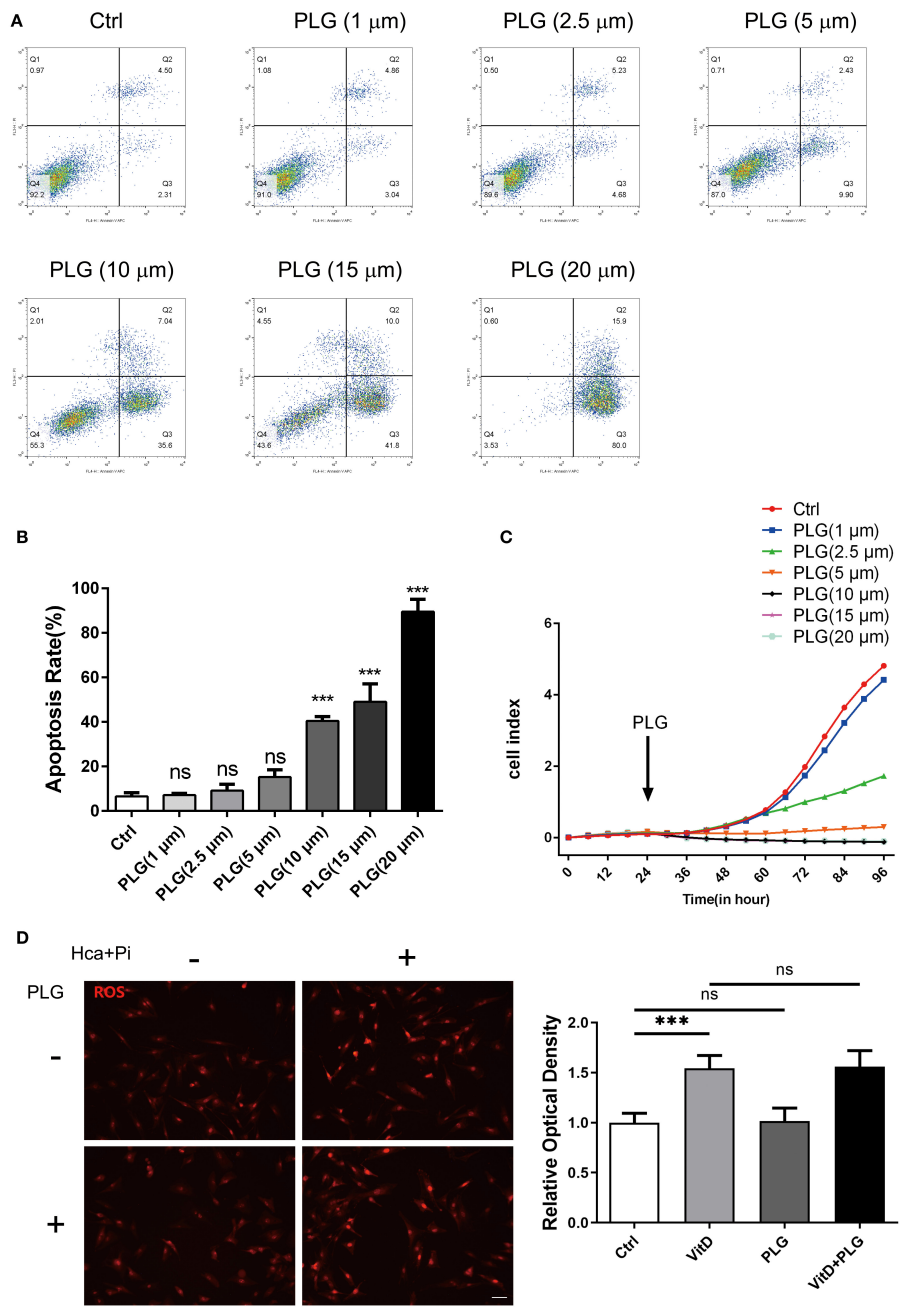
The effect of PLG on the apoptosis and proliferation of RVSMCs. **(A)** Measurement of apoptosis by flow cytometry. RVSMCs were treated with different concentrations of PLG for 72 h. **(B)** Statistical analysis of the apoptosis ratio. **(C)** Cell proliferation as determined by the real-time cell analysis index (***P < 0.001 vs. control). **(D)** Representative images of DHE staining for ROS in RVSMCs (scale bars = 50 μm; *n* = 6 for each group; ***P < 0.001 indicate significant differences between the indicated columns).

First, we used flow cytometry to detect the effect of PLG on VSMCs apoptosis. Compared to the control group, there was no difference in PLG treatment group within the 1–5 μM concentration range (Figures 1A,B). When the concentration range of PLG was 10–20 μM, PLG promoted the apoptosis of VSMCs and showed a certain dose correlation. Next, we used a RTCA analyser to determine the cytotoxicity of PLG. Compared to the control group, no obvious cell death or damage was observed after VSMCs were incubated with 1 to 2.5 μM PLG for 72 h. These results indicated that the concentration of PLG at 1–2.5 μM does not affect the survival and apoptosis of VSMCs (Figure 1C). PLG has been demonstrated as an inducer of reactive oxygen species (ROS) (15). PLG can selectively kill cancer cells, but not normal cells, by accumulation of ROS (16). To assess the effect of PLG on ROS levels in VSMCs, we used Dihydroethidium to detect ROS levels in VSMCs after 1-day treatment with calcification medium and found that the level of ROS in cells did not increase under 2.5 μM PLG treatment (Figure 1D). Therefore, we used PLG at a concentration of 2.5 μM in subsequent experiments.

### Piperlongumine Inhibits VSMCs Calcification Induced by High Calcium/Phosphate Medium

**Figure 2:**
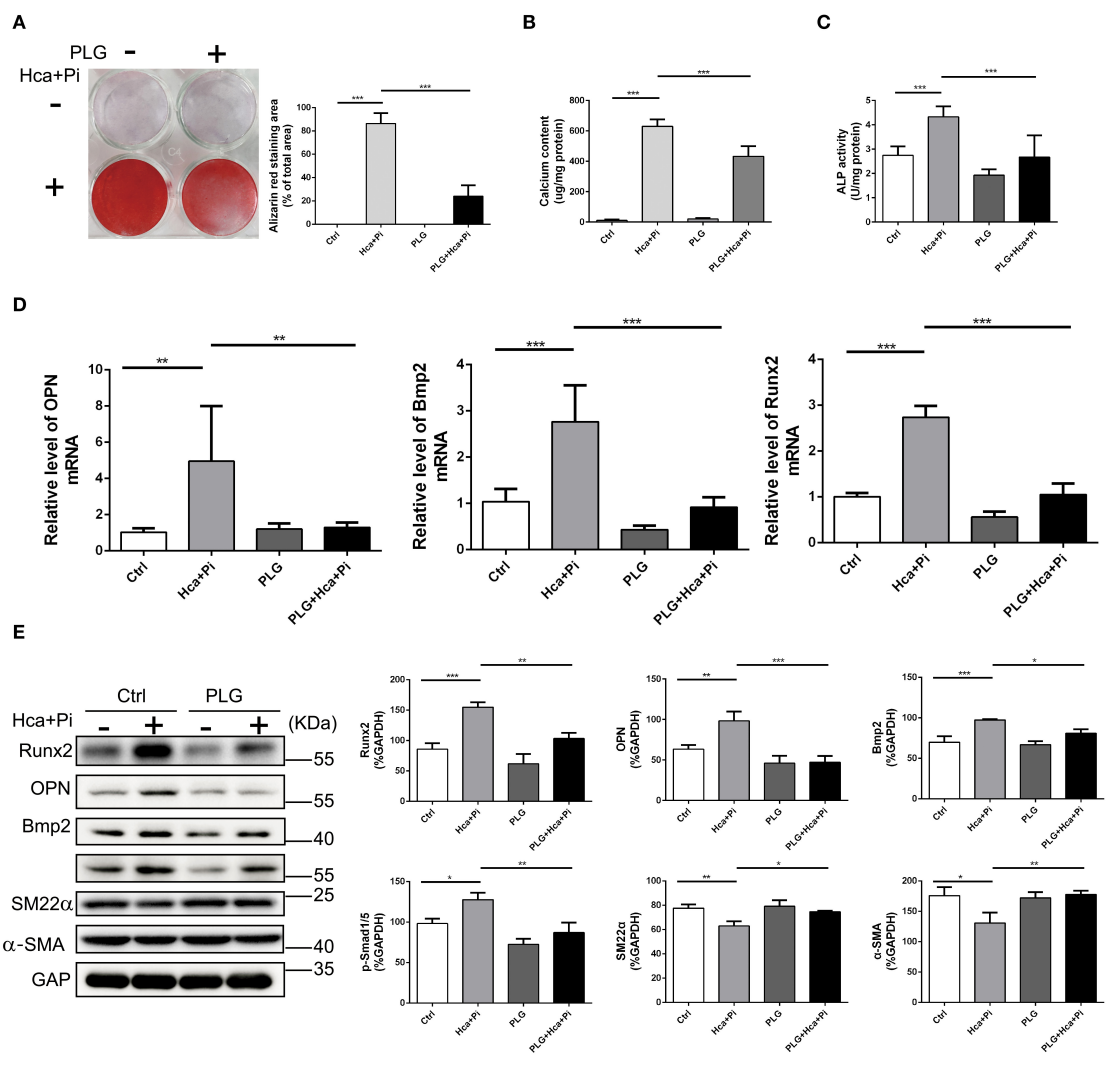
PLG inhibits VSMCs calcification induced by high calcium/phosphate medium. **(A)** Alizarin Red staining of cellular calcium deposition. The histogram on the right is a statistical analysis of the Alizarin Red staining area. **(B)** Calcium content was measured with the QuantiChrom™ Calcium Assay Kit. **(C)** ALP activity was measured with the LabAssay™ ALP Kit. **(D)** RVSMCs were treated with PLG (2.5 μM) and high calcium/phosphate. The mRNA levels of Runx2, OPN, and Bmp2 were measured by qRT-PCR (*n* = 6 for each group). **(E)** The protein levels of Runx2, OPN, Bmp2, p-Smad1/5, SM22α, and α-SMA were measured by western blotting. The relative protein levels were normalized to the level of GAPDH. The data are shown as the mean ± standard error of triplicate sets and are representative of three independent experiments (*P < 0.05, **P < 0.01, and ***P < 0.001 indicate significant differences between the indicated columns).

As mentioned above, VSMCs play important roles in vascular calcification, and they constitute the main cell type in vascular calcification. Therefore, we extracted primary vascular smooth muscle cells and used high calcium/phosphate medium to induce calcification of VSMCs. Alizarin red staining and the cellular calcium content showed that high calcium/phosphate medium significantly increased calcium deposition in the VSMCs but that PLG blocked calcium deposition in cells (Figures 2A,B). The measured ALP activity in the VSMCs demonstrated that high calcium/phosphate medium increased the ALP activity and that this increase in ALP activity was significantly inhibited by PLG (Figure 2C). To measure the effect of PLG on VSMCs calcification at the molecular level, cells cultured in normal medium and high calcium/phosphate medium were treated with or without PLG, and the expression levels of Runx2, OPN and Bmp2, which are related to osteogenic differentiation, were measured. High calcium/phosphate medium increased the protein and mRNA expression of Runx2, OPN and Bmp2, but PLG reduced the high calcium-/phosphate- induced increases in Runx2, OPN and Bmp2 protein and mRNA expression (Figures 2D,E). In summary, our data indicated that PLG attenuates high calcium- and phosphate- induced VSMCs calcification.

### Piperlongumine Inhibits Vit D-Induced Aortic Calcification in C57BL/6 Mice

**Figure 3:**
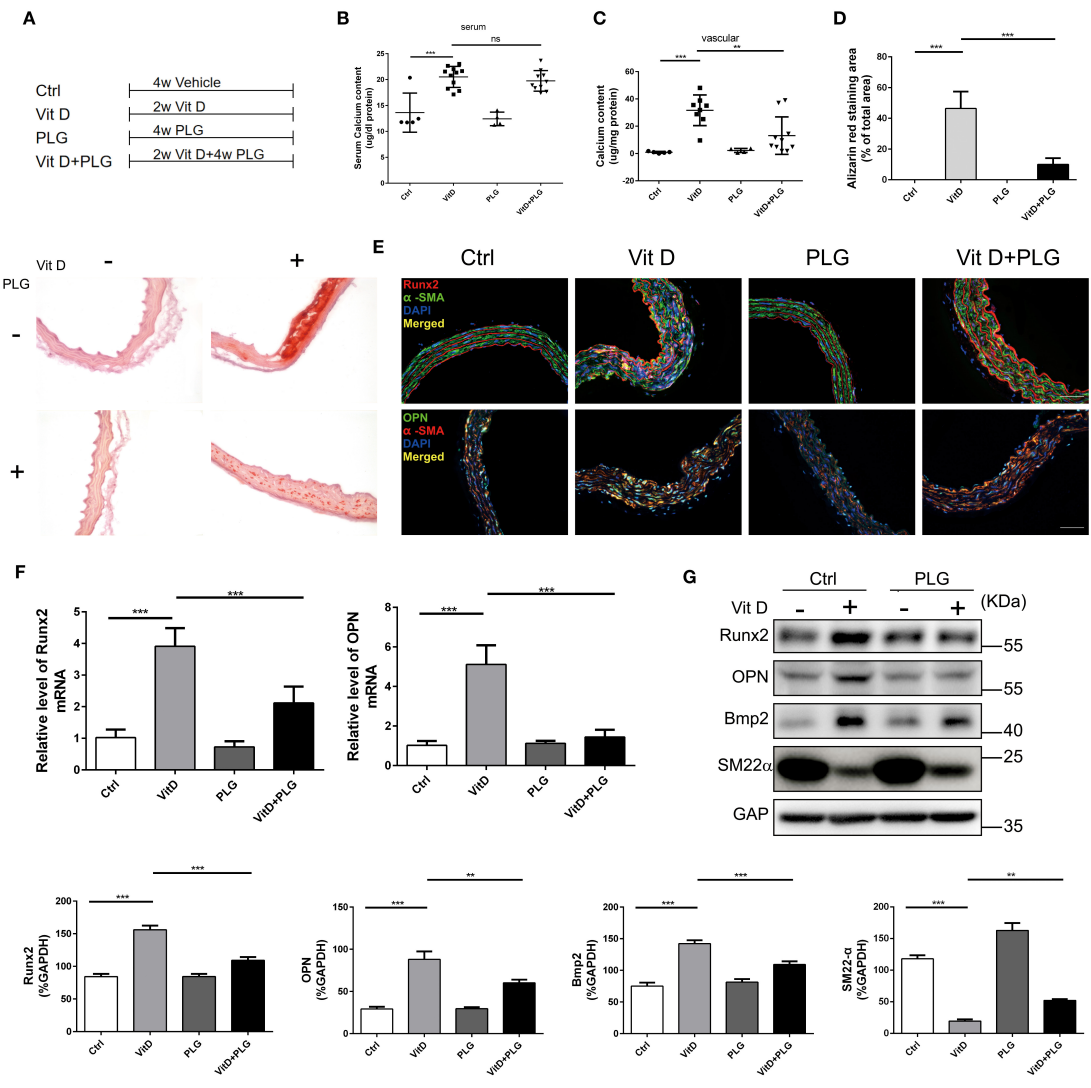
PLG inhibits aortic calcification induced by vitamin D in mice. **(A)** Four groups of C57BL/6 mice were subjected to the indicated treatments for 4 weeks (Vit D, 8.75 mg/kg/day and PLG, 5 mg/kg body weight every 2 days). **(B,C)** Serum calcium content and aortic calcium deposition were measured with a QuantiChrom™ calcium assay kit. **(D)** Calcium deposition was assessed by Alizarin red staining in mouse aortas (scale bars = 20 μm; *n* = 6 for each group). The histogram on the right is the quantitative analysis of the Alizarin red-stained areas. **(E)** Representative images of immunofluorescence staining of Runx2 and OPN in mouse aortas (scale bars = 20 μm, *n* = 6 for each group). **(F)** The mRNA levels of Runx2 and OPN in mouse aortas were measured by qRT-PCR (*n* = 6 for each group). **(G)** The protein levels of Runx2, OPN, Bmp2, and SM22α in mouse aortas were assessed by western blotting. The relative protein levels were normalized to the level of GAPDH. The data are shown as the mean ± standard error of triplicate sets and are representative of three independent experiments (**P < 0.01, and ***P < 0.001 indicate significant differences between the indicated columns).

To further explore the therapeutic potential of PLG on vascular calcification, we established an animal model of Vit D-induced aortic calcification in mice. We allocated the animals into four treatment groups as indicated in Figure 3A. Two weeks after intraperitoneal injection of Vit D, PLG significantly reduced the calcium content in the aorta, and there was no significant difference in serum calcium content (Figures 3B,C). Alizarin red staining showed that compared to the control mice, Vit D-treated mice exhibited obvious calcium deposits in the aorta, while PLG- treatment had a certain inhibitory effect on calcium deposition (Figure 3D). We also measured the mRNA and protein expression levels of the Runx2, Bmp2, and OPN osteogenic markers *in vivo*. Consistent with the *in vitro* results, the western blotting and qRT-PCR results showed that the expression of Runx2, Bmp2, and OPN increased in the aorta of the mice after Vit D treatment and that PLG inhibited this increase (Figures 3F,G). This conclusion was confirmed by immunofluorescence staining of mouse aortas (Figure 3E). These findings confirmed that PLG has a certain inhibitory effect on smooth muscle calcification both *in vitro* and *in vivo*.

### Piperlongumine Upregulates P53 Signaling in VSMCs and C57BL/6 Mice

**Figure 4:**
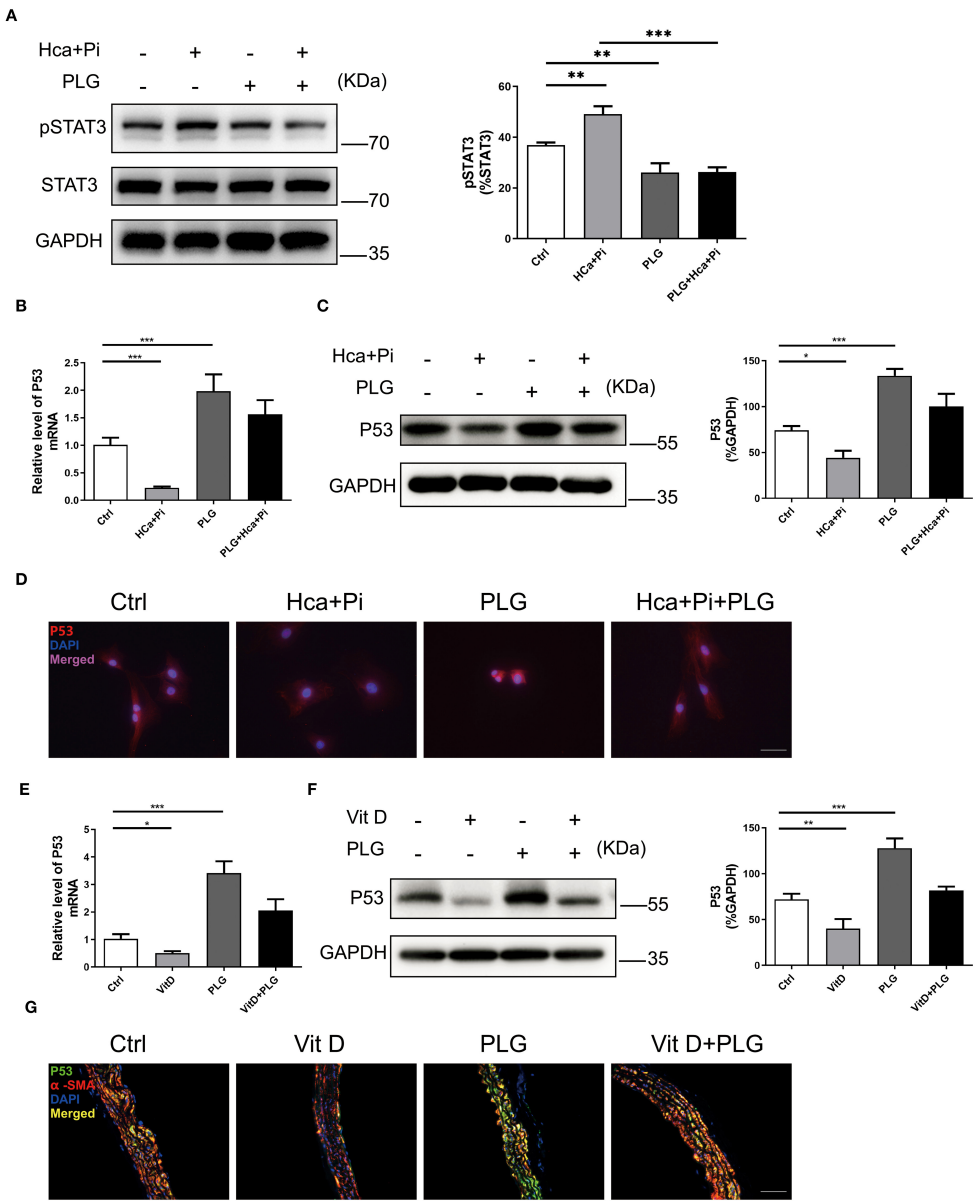
PLG upregulates P53 signaling *in vivo* and *in vitro*. **(A)** The protein levels of pSTAT3 in the RVSMCs were measured by western blotting. The relative protein levels were normalized to the level of STAT3. The data are shown as the mean ± standard error of triplicate sets and are representative of three independent experiments. **(B)** The mRNA levels of P53 in the RVSMCs were measured by qRT-PCR (*n* = 6 for each group). **(C)** The protein levels of P53 in the RVSMCs were measured by western blotting. The relative protein levels were normalized to the level of GAPDH. The data are shown as the mean ± standard error of triplicate sets and are representative of three independent experiments. **(D)** Representative images of immunofluorescence staining for P53 in RVSMCs (scale bars = 50 μm; *n* = 6 for each group; *P < 0.05, **P < 0.01, and ***P < 0.001 indicate significant differences between the indicated columns). **(E)** The mRNA levels of P53 in mouse aortas were measured by qRT-PCR (*n* = 6 for each group). **(F)** The protein levels of P53 in mouse aortas were measured by western blotting. The relative protein levels were normalized to the level of GAPDH. The data are shown as the mean ± standard error of triplicate sets and are representative of three independent experiments. **(G)** Representative images of immunofluorescence staining for P53 in mouse aortas (scale bars = 20 μm; *n* = 6 for each group; *P < 0.05, **P < 0.01, and ***P < 0.001 indicate significant differences between the indicated columns).

PLG has antitumour activity and significantly increases the expression of wild-type P53 (17). Previous study has revealed that PLG directly inhibited binding of Signal transducer and activator of transcription 3 (STAT3) to its phosphotyrosyl peptide ligand and constitutive STAT3 phosphorylation, and modulated expression of multiple STAT3-regulated genes (18). Activated STAT3 can bind to the P53 promoter, then inhibit P53 expression in a STAT3-dependent manner (19, 20). We hypothesize that P53 activation by PLG in VSMCs is mediated by decreased activation of STAT3. Western blotting showed that high calcium/phosphate treatment increased the level of pSTAT3 in VSMCs, and PLG significantly decreased the phosphorylation of STAT3 compared to the control group (Figure 4A). Then we measured the P53 expression level in the experimental cells and animals. Western blotting and qRT-PCR analyses showed that high calcium/phosphate treatment reduced the P53 expression level in VSMCs and that PLG significantly increased the P53 expression level compared to the control group. PLG also weakened the inhibitory effect of high calcium/phosphate treatment on P53 (Figures 4B,C). We performed immunofluorescence staining of VSMCs to observe the expression of P53, and similar results were obtained (Figure 4D). We also measured the protein and mRNA expression of P53 in the aorta of the calcification model mice. Similarly, the expression of P53 in the Vit D group was significantly reduced, and PLG treatment effectively increased the expression of P53 in the aorta (Figures 4E,F). P53 immunofluorescence staining of mouse aorta also demonstrated the same results (Figure 4G). These results showed that PLG upregulated the expression of P53 during vascular calcification by reducing STAT3 phosphorylation.

### Piperlongumine Exerts Anti-calcification Functions by Upregulating the P53 Signaling Pathway

**Figure 5:**
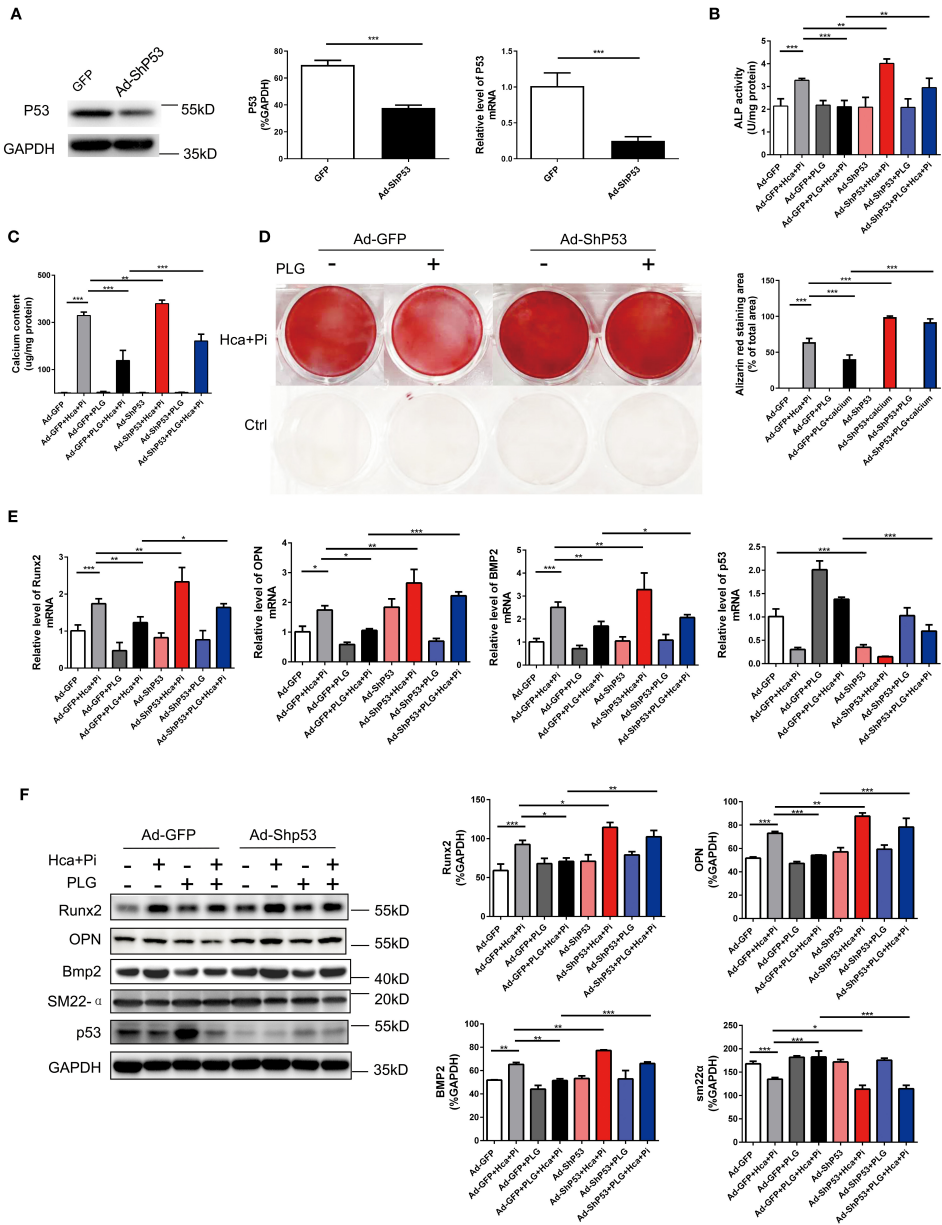
PLG exerts anti-calcification functions by upregulating the P53 signaling pathway. **(A)** The efficacy of Ad-ShP53 for the knockdown of P53 was assessed by western blotting and qRT-PCR (*n* = 6). The relative protein levels of P53 were normalized to the level of GAPDH. The data are shown as the mean ± standard error of triplicate sets and are representative of three independent experiments. **(B)** RVSMCs were treated with PLG (2.5 μM) and high calcium/phosphate. After knocking down P53 with P53 ShRNA, ALP activity was measured with the LabAssay™ ALP Kit. **(C)** Calcium content was measured with the QuantiChrom™ Calcium Assay Kit. **(D)** Alizarin red staining was used to evaluate cellular calcium deposition. **(E)** The mRNA levels of Runx2, OPN, Bmp2, and P53 in RVSMCs were measured by qRT-PCR after knocking down P53 (*n* = 6 for each group). **(F)** The protein levels of Runx2, OPN, Bmp2, SM22α, and P53 were measured by western blotting. The relative protein levels were normalized to the level of GAPDH. The data are shown as the mean ± standard error of triplicate sets and are representative of three independent experiments (*P < 0.05, **P < 0.01, and ***P < 0.001 indicate significant differences between the indicated columns).

To confirm that PLG acts via P53 in the high calcium- and phosphate-induced calcification models, we downregulated the expression of P53 through adenoviral transduction. We first verified the efficiency of adenovirus transduction by qRT-PCR and western blotting analyses, which indicated that Ad-ShP53 transduction successfully knocked down the expression of P53 (Figure 5A). Subsequently, knocking down P53 after transduction of Ad-ShP53 significantly increased the calcium deposition and ALP activity of the VSMCs, and it partially reversed the protective effect of PLG on VSMCs calcification (Figures 5B,C). Alizarin red staining of the intracellular calcium deposition of the VSMCs also showed the same phenomenon (Figure 5D). Transduction of Ad-ShP53 increased the mRNA and protein expression of osteogenic markers, including Runx2, Bmp2, and OPN, and it reversed the inhibitory effect of PLG on Runx2, Bmp2, and OPN (Figures 5E,F). These findings demonstrated that PLG reduces the calcification in VSMCs by regulating P53, indicating that P53 plays an important role in the calcification of VSMCs.

### Piperlongumine Promotes PTEN Expression by Increasing P53 Signaling

**Figure 6:**
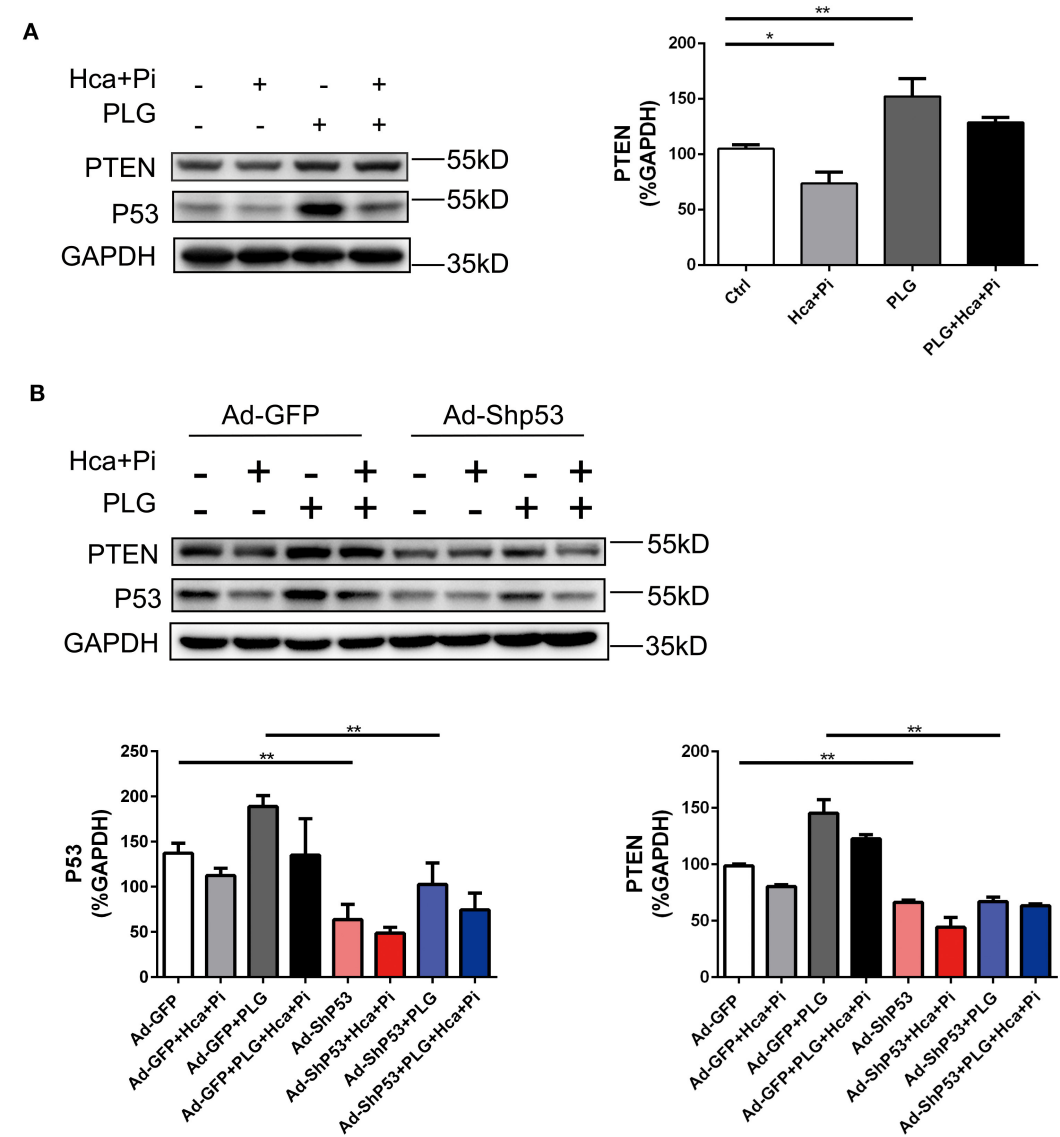
PLG promotes PTEN expression by increasing P53 signaling. **(A)** The relative protein levels of PTEN were normalized to the level of GAPDH. The data are shown as the mean ± standard error of triplicate sets and are representative of three independent experiments. **(B)** RVSMCs were treated with PLG (2.5 μM) and high calcium/phosphate. After verifying the efficacy of shRNA for knockdown of P53, the relative protein levels of PTEN in the RVSMCs were normalized to the level of GAPDH. The data are shown as the mean ± standard error of triplicate sets and are representative of three independent experiments (*P < 0.05, **P < 0.01 indicate significant differences between the indicated columns).

PTEN is a protein/lipid phosphatase that was originally discovered as a tumor suppressor (21). PTEN is induced by P53 in the early and late stages of cell response, and PTEN and P53 interact (22, 23). A previous study reported that SMC-specific PTEN deletion leads to continuous activation of AKT, which upregulates Runx2 and promotes VSMCs calcification and arterial calcification. Knocking out Runx2 prevents PTEN deletion-induced calcification, indicating that PTEN deletion promotes Runx2-dependent smooth muscle calcification (24). We used western blotting analysis to detect the expression of PTEN under different treatment conditions and found that the expression of PTEN was consistent with the trend of P53. In the case of high calcium/phosphate treatment, the expression of P53 was decreased, while PLG increased the expression of PTEN. After knocking down P53 with adenovirus, the expression of PTEN also decreased. Therefore, we concluded that PLG exerted its anti-calcification effect in smooth muscle by affecting P53/PTEN (Figure 6).

These experiments demonstrate that PLG attenuates arterial calcification by upregulating the P53/PTEN signaling pathway and that this inhibitory effect on calcification can be blocked by P53 knockdown.

## Discussion

The main findings of the present study indicated that (1) PLG is a promising natural herbal extract for the management of vascular calcification and that (2) PLG attenuates high calcium- and phosphate-induced vascular calcification by preserving P53/PTEN signaling in VSMCs.

Because the existing treatment methods for vascular calcification have certain limitations, finding effective treatment methods and therapeutic drugs for vascular calcification is of great importance. Here, we found that PLG inhibited the vascular calcification induced by a high calcium/phosphate medium and Vit D. The reported pharmacological effects of PLG include cytotoxicity, anti-angiogenesis, and anti-atherosclerosis (25). The direct cytotoxic activity of PLG against tumor cell lines has been described in many studies. The IC50 value of PLG for normal cells is higher than that for tumor cells, and PLG shows low toxicity in normal aortic endothelial cells (IC_50_ > 15 μM). PLG has selective cytotoxicity in cancer cells but weak activity in normal cells (25). The dose of PLG used in the present *in vitro* studies (2.5 μM) was far below the reported toxic dose, thereby suggesting PLG safety for long-term at this low dose. A previous study has shown that a 50 mg/kg dose of PLG leads to minor organ toxicity in healthy mice, as characterized with mild and reversible kidney effects (26). Our data showed no systemic toxicity of the lower dose of PLG (5 mg/kg body weight every 2 days) in C57BL/6 mice. There was no significant difference in survival rate between the control group and the PLG group. According to the study by Son et al., treatment with l and 5 μM PLG significantly inhibits plaque formation in partial ligated carotid arteries of ApoE-KO mice, and cell viability is not significantly altered when VSMCs are treated with PLG (up to 10 μM) (11). In the present study, we found that a low concentration of PLG did not affect VSMCs proliferation or apoptosis but had significant anti-calcification effects. Our results suggested that long-term use of PLG at a low dose was safe and effective for prevention of vascular calcification.

P53, a tumor suppressor gene, has an important regulatory role in cell proliferation, apoptosis, cell cycle regulation and DNA damage repair. Studies have found that P53 also has a regulatory role in osteoblast differentiation, bone formation, and osteoclast-dependent osteoclast differentiation. P53-deficient mice show increased bone formation and osteosclerosis phenotypes, and P53-deficient osteoblasts have an increased proliferation rate (27–29). Deletion of MDM2, an inhibitor of P53, in osteoblast lineage cells leads to increased P53 production, which in turn inhibits bone organogenesis and homeostasis (30). Runx2 is a master transcription factor involved in bone formation and vascular calcification, and P53 can interact with Runx2 during osteogenic differentiation (29). The loss of P53 leads to abnormal expression of Runx2 in osteosarcoma, which further leads to bone matrix remodeling and tissue calcification (31). Osteosarcoma cells with P53 deletion have higher levels of Runx2 and faster osteogenic differentiation than those with wild-type P53 (28). Teniposide, a DNA topoisomerase II inhibitor, reduces atherosclerosis and vascular calcification in ApoE deficient mice by inactivating Bmp2/Runx2 axis in a P53-dependent manner (32). These findings suggest that P53 may inhibit the osteogenic differentiation of VSMCs by inhibiting Runx2 function. In tumor cells, PLG decreases topoisomerase II and Bcl-2 expression, resulting in increased P53 expression, which promotes cell apoptosis in a concentration- and time-dependent manner (33, 34). The effective PLG concentration used (10 to 15 μM) to induce apoptosis in tumor cells increases P53 by three- to four-fold compared to the level in control cells (35). In the present study, the PLG-induced increase in P53 expression was much lower than the level of P53 that can cause apoptosis. We used a low dose of PLG (2.5 μM) that had no effect on cell apoptosis but increased P53 expression, indicating the differential regulation of P53 in different cells. Moreover, P53 regulates PTEN in the early and late stages of cell response, and there PTEN and P53 interact (22, 23). PTEN deletion promotes Runx2-dependent VSMCs calcification, and the inhibition of PTEN enhances intramembranous and late endochondral fracture healing (28, 36). In our study, we found that PLG exerted anti-calcification functions by upregulating the P53/PTEN signaling pathway.

Some extracts from natural herbs, including Ginkgo and Radix Puerariae, have protective effects against vascular calcification (37, 38). Ginkgo biloba extract (GBE) is produced from Ginkgo leaves and contains several biologically active substances, making it difficult to isolate the effects of the individual biologically active substances. In a recent National Toxicology Program study, chronic exposure of B6C3F1/N mice to GBE resulted in a high incidence of hepatocellular carcinomas (39). Puerarin is a phytoestrogen extracted from Radix Puerariae that attenuates the osteoblastic differentiation of VSMCs through the ER/PI3K-Akt signaling pathway. However, poor water solubility and limited oral bioavailability limit the use of puerarin. Moreover, the use of puerarin is limited by some severe adverse events, including intravascular haemolysis (40). PLG is a biologically active alkaloid extracted from *Piper longum* L. with a specific chemical structure. *Piper Longum* L. belongs to the Piperaceae family. In Eastern countries, *Piper Longum* L. is often used as a seasoning due to its special pepper-like flavor. *Piper Longum* L. has long been a popular condiment for traditional Oriental foods, such as hot pot and braised pork. As a compound extracted from *Piper Longum* L., PLG shows great oral bioavailability in mice, and the bioavailability of PLG following oral administration at 5 mg/kg is 76.39% (25). Overall, our experimental data and the characteristics of PLG indicate that PLG might be a promising natural compound for the management of vascular calcification.

## Data Availability Statement

The original contributions generated for the study are included in the article/Supplementary Material, further inquiries can be directed to the corresponding author/s.

## Ethics Statement

The animal study was reviewed and approved by The Institutional Animal Care and Use Committee of Nanjing Medical University (Nanjing, China).

## Author Contributions

WSu and XK developed the conception and design of the study. WSh, JiL, JuL, and MQ performed experiments and collected data. WSh, JuL, MQ, JG, and YL analyzed, interpreted, and discussed data. WSh and MQ wrote the manuscript. All authors contributed to the article and approved the submitted version.

## Conflict of Interest

The authors declare that the research was conducted in the absence of any commercial or financial relationships that could be construed as a potential conflict of interest.
