# Supplementary material for: Piperlongumine Attenuates High Calcium/Phosphate-Induced Arterial Calcification by Preserving P53/PTEN Signaling
Source: Front Cardiovasc Med. 2021 Feb 10;7:625215. doi: 10.3389/fcvm.2020.625215 (PMC7903972; doi:10.3389/fcvm.2020.625215)
Supplement: Supplementary file 1 [file Data_Sheet_1.PDF]

Supplementary Information

Supplementary Materials and Methods

Ethics statement

The Institutional Animal Care and Use Committee of Nanjing Medical University (Nanjing, China) approved all the animal protocols. All the procedures involving animals were conducted in accordance with the Guide for the Care and Use of Laboratory Animals published by the National Institutes of Health (no. 85-23; revised 1996), and the study protocol was approved by the Institutional Animal Care and Use Committee (IACUC) of Nanjing Medical University (Nanjing, China; Approval No. IACUC-1710005).

Determination of PLG concentration changes in serum

Wild type mice (males, 8 weeks old, 23–26 g, n = 6) were peritoneally injected with PLG (5 mg/kg) and serum was collected at different time points. Liquid chromatography-mass spectrometry was used to measure the PLG concentration in serum.

Supplementary Figures and Figure Legends

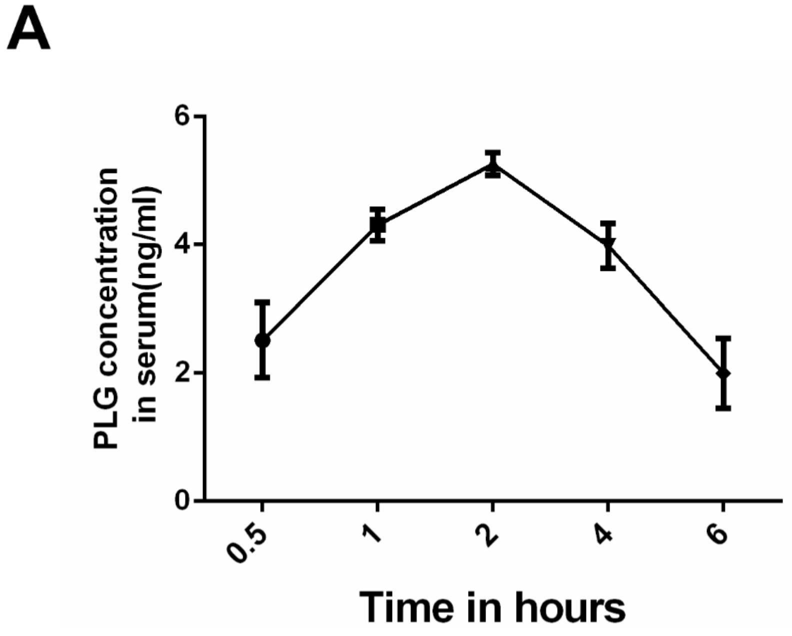

Supplementary Figure 1. Determination of PLG concentration changes in serum using liquid chromatography-mass spectrometry (n=6).

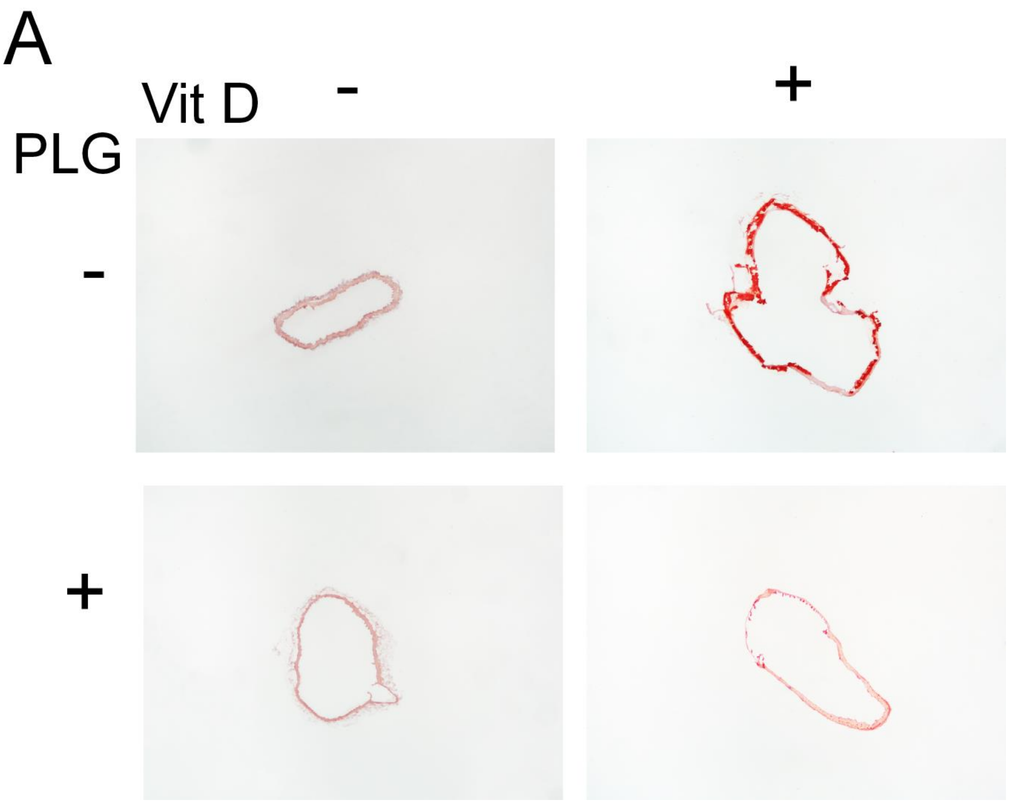

Supplementary Figure 2. PLG inhibits aortic calcification induced by vitamin D in mice.(A) Calcium deposition was assessed by Alizarin red staining in mouse aortas (scale bars = 50 μm; n = 6 for each group).

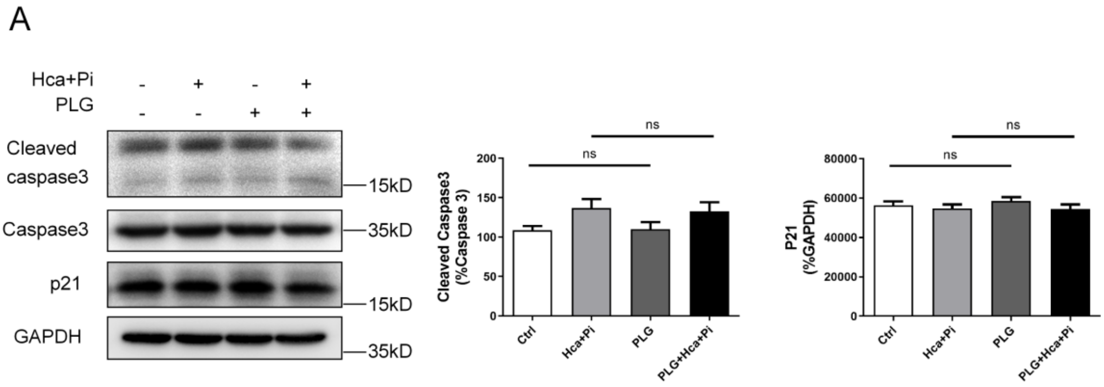

Supplementary Figure 3. The effect of PLG on the apoptosis of VSMCs. (A) The protein levels of Cleaved Caspase 3, Caspase 3 and P21 were measured by western blotting. The data are shown as the mean ± standard error of triplicate sets and are representative of three independent experiments (*P < 0.05, **P < 0.01, and ***P < 0.001 indicate significant differences between the indicated columns).

A

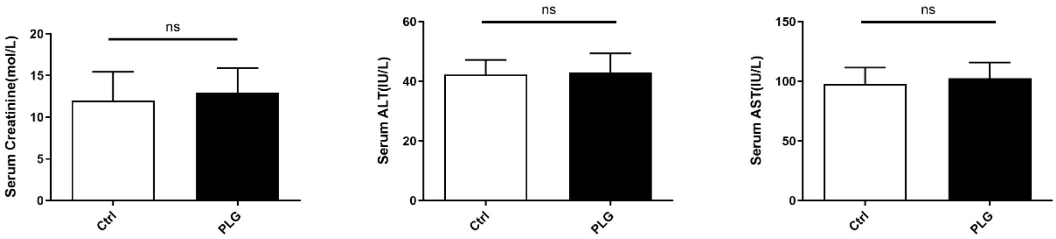

Supplementary Figure 4. PLG treatment showed no significant toxic effects. (A)Serum AST, ALT and creatinine were measured.(n=6 for each group)
